# Thymic development of human natural killer T cells: recent advances and implications for immunotherapy

**DOI:** 10.3389/fimmu.2024.1441634

**Published:** 2024-08-29

**Authors:** Daniel G. Pellicci, Naeimeh Tavakolinia, Louis Perriman, Stuart P. Berzins, Christopher Menne

**Affiliations:** ^1^ Murdoch Children’s Research Institute, Melbourne, VIC, Australia; ^2^ Department of Microbiology and Immunology, University of Melbourne, Melbourne, VIC, Australia; ^3^ Department of Paediatrics, University of Melbourne, Melbourne, VIC, Australia; ^4^ Fiona Elsey Cancer Institute, Ballarat, VIC, Australia; ^5^ Federation University Australia, Ballarat, VIC, Australia

**Keywords:** unconventional T cells, unconventional T cell development, cytokines, cytotoxic granules, immunotherapy

## Abstract

Invariant natural killer T (iNKT) cells are a subset of lipid-reactive, unconventional T cells that have anti-tumor properties that make them a promising target for cancer immunotherapy. Recent studies have deciphered the developmental pathway of human MAIT and Vγ9Vδ2 γδ-T cells as well as murine iNKT cells, yet our understanding of human NKT cell development is limited. Here, we provide an update in our understanding of how NKT cells develop in the human body and how knowledge regarding their development could enhance human treatments by targeting these cells.

## Introduction

T cells are highly specialized lymphocytes that undergo a complex maturation and differentiation program that gives rise to distinct T-cell lineages with unique effector functions. Conventional T cells that recognize peptide antigens presented by the major histocompatibility complex (MHC) undergo well-characterized processes of antigen receptor rearrangement followed by positive and negative selection in the thymus. They typically exit the thymus as naive progenitors that can be primed upon primary antigen encounter in the secondary lymphoid organs, which confer functional maturity and effector subset differentiation.

The thymus also supports the development of several populations of unconventional T cells that appear to undergo a decidedly distinct maturation process. Unconventional T cells typically recognize non-peptide antigens presented by distinct antigen-presenting molecules. CD1d molecules present lipid antigens to natural killer T (NKT) cells, MR1 presents microbial riboflavin derivatives to mucosal-associated invariant T (MAIT) cells, and human gamma delta (γδ) T cells are potently activated by a wide range of ligands, e.g., Vδ2+ T cells react to phosphoantigen-bound butyrophilin molecules. NKT cells are defined by their reactivity to CD1d-restricted lipid antigens, and humans contain two main subsets of NKT cells, called type 1 or invariant NKT (iNKT) cells and type 2, or diverse NKT cells (1).

Human iNKT cells express a semi-invariant alpha beta T-cell receptor (αβ-TCR) composed of a Vα24Jα18 alpha chain paired to Vβ11 that recognizes the prototypic ligand alpha-galactosylceramide (αGalCer) presented by CD1d. Diverse NKT cells express diverse TCRs that can also recognize lipid antigens presented by CD1d, but do not recognize αGalCer (1). αGalCer loaded CD1d tetramers and monoclonal antibodies specific to human TCR chains of iNKT cells are widely used tools to stringently identify iNKT cells in health and disease settings (2–6). Diverse NKT cells are considered to be slightly more abundant in humans, but the lack of specific reagents to identify them has hindered their detailed analysis (1). Hence, this review will focus on human iNKT cells and how our knowledge of their development could enhance human immunotherapies that target these cells.

## Human iNKT cells

Human iNKT cells display great variability in responding to activation stimuli (7, 8). Remarkably, they exhibit dual functionality via differential secretion of pro- and anti-inflammatory cytokines, and they can also display direct cytotoxicity via the release of lytic granules or via death receptor ligation (9–15). They play important roles in regulating immune responses associated with tumor surveillance, infectious diseases, autoimmunity, and graft versus host disease (GvHD), and are increasingly seen as attractive therapeutic targets to prevent human diseases (16, 17).

In contrast to mouse iNKT cells that represent ~1% of blood and up to 45% of T cells in the liver, human iNKT cells are much rarer. They account for approximately 0.1% of T cells in peripheral blood [reviewed in (1)], and their numbers are only slightly higher in organs such as the liver and spleen, with the exception of the omentum and adipose tissue where they constitute ~10% and 1% of T cells, respectively (1, 18, 19). iNKT cells can be activated in a TCR-dependent manner by recognition of lipid antigens presented by CD1d or in a TCR-independent manner by cytokines such as IL-12 and IL-18 (8). Both pathways are important for their dual functionality.

## NKT cell heterogeneity in humans and mice

**Figure 1:**
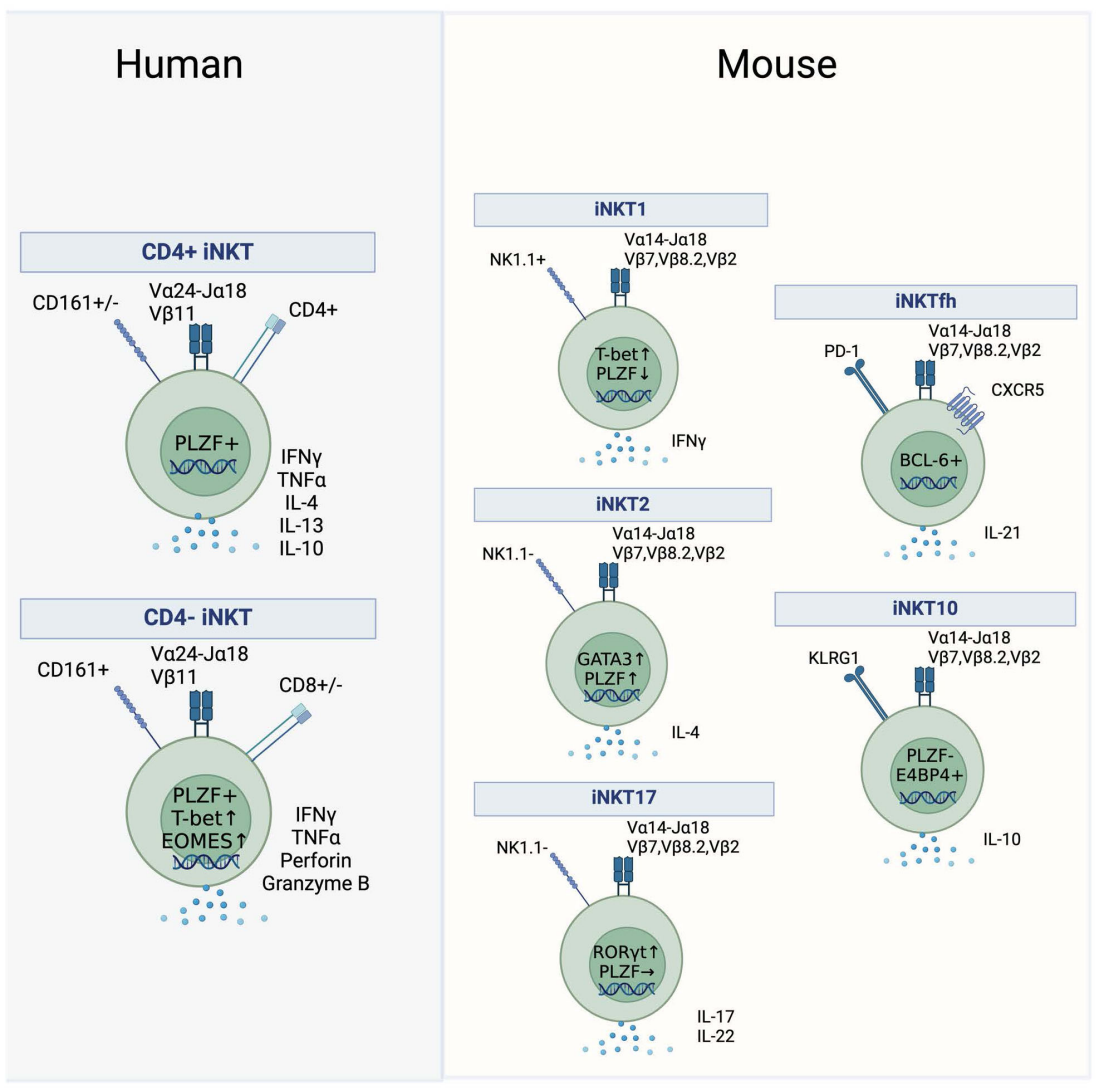
Human and mouse iNKT subsets. Subsets of iNKT cells in humans (left) and mice (right). Key surface markers, cytokines and transcription factors that define the subsets are shown.

In humans, iNKT cells can be broadly grouped into three main subsets defined by differential expression of CD4 and CD8 (**Figure 1**). Whereas mice only have CD4+ and CD4−CD8− double-negative (DN) iNKT subsets, humans contain an additional subset of CD8+ iNKT cells (20–22). Generally, human CD4+ iNKT cells differ functionally from their CD4− counterparts. CD4+ iNKT cells exhibit a T helper, TH0 cytokine signature and can simultaneously secrete IFNγ and IL-4, whereas CD4− iNKT cells lack IL-4 secretion, exhibit an IFNγ-positive T_H_1 cytokine profile, express several NK receptors, and can also mediate direct cytotoxicity (**Figure 1**) (23, 24). Minor differences exist between human DN and CD8 iNKT cells in their T_H_1 cytokine secretion and cytotoxicity (10, 13, 24, 25). Other variations of human iNKT cells have been described, marked by the expression of CD161 or CD62L, although their significance is not well defined (23). Single-cell RNA sequencing of unstimulated and stimulated human iNKT cells revealed additional clusters of functionally diverse iNKT cells, while also corroborating well-established functional differences between CD4+ and CD4− subsets (26).

Effector subsets in mice are not grouped according to the expression of CD4 but are rather defined by the expression of transcription factors and their ability to secrete cytokines. After thymic development in the mouse, functional iNKT subsets diversify into three separate lineages termed iNKT1, iNKT2, and iNKT17, analogous to conventional TH1, TH2 and TH17 subsets (**Figure 1**). NKT1 cells are low in PLZF and high in Tbet and secrete IFNγ, NKT2 cells express high levels of PLZF and GATA3 and secrete IL-4, whereas NKT17 cells have high expression levels of RORγt coupled with intermediate PLZF expression and secrete IL-17 (**Figure 1**) (27–29). It is unclear if human equivalents of these subsets exist, as human iNKT cells appear to be polarized towards a T_H_1 or mixed T_H_0 functionality while their ability to produce IL-17 *ex vivo* is more controversial (30–33). Furthermore, even though human iNKT cells express PLZF and the protein levels of Tbet and EOMES vary between the CD4+ and CD4− subset, the classification of human iNKT cells based on the expression of key transcription factors has not been investigated in detail (34–36).

Besides the well-defined murine NKT1, NKT2, and NKT17 and the human CD4+ and CD4− subsets, there are reports of additional iNKT subsets. Immunoregulatory iNKT cells capable of secreting IL-10 were described in mice and in human PBMCs (37). In mice, this subset is termed NKT10 cells, and these cells are enriched in adipose tissue (**Figure 1**) (38). They are characterized by the absence of PLZF and by the expression of the transcription factor E4BP4 (38). An additional immunoregulatory, suppressive iNKT subset is induced in mice and humans upon stimulation in the presence of TGF-β or rapamycin resulting in the expression of FOXP3 (39, 40). In mice, the emergence of follicular helper NKT (NKTfh) cells was demonstrated following αGalCer administration (**Figure 1**). This subset closely resembled conventional Tfh cells as it was dependent on the expression of BCL-6, expressed PD-1 and CXCR5, and was able to induce germinal centers via IL-21 signaling (41). An analogous human subset of PD-1 and CXCR5 co-expressing iNKT cells was discovered in human tonsils (41).

## Development of mouse iNKT cells

We have previously reviewed the development of mouse iNKT cells, including a detailed description of key signaling molecules, transcription factors, and microRNAs that regulate this process (27). Early studies by us and others suggested that mouse iNKT cells develop in a linear manner, via a 4-stage maturation pathway characterized by the differential expression of CD24, CD44, and NK1.1, with fully mature cells expressing NK1.1 (27, 42, 43). However, subsequent studies revealed that mature subsets of iNKT2 and iNKT17 cells were present within mouse thymus that lacked the expression of NK1.1 (29, 44). Thus, these later studies indicated that thymic iNKT cells developed via lineage diversification, from a common CD24hi, CD44lo, NK1.1− iNKT0 progenitor into distinct mature iNKT1, iNKT2, and iNKT17 subsets. More recently, single-cell sequencing analysis suggested that mouse iNKT2 cells represent a branching point for the generation of iNKT1 and iNKT17 cells, and that iNKT1 cells upregulate NK1.1 upon final maturation (45).

PLZF is described to be the master regulator of unconventional T-cell development, and PLZF is absolutely required for development of functionally mature subsets of mouse iNKT cells and MAIT cells (27, 35, 36, 46, 47). The expression of PLZF in mouse iNKT cells is regulated by the transcription factors Egr2 and Runx1 (48, 49). PLZF expression is first detected in mouse NKT0 progenitors and high levels of PLZF are maintained by iNKT2 cells, whereas PLZF is downregulated in mature subsets of mouse iNKT1 and iNKT17 cells (27, 29, 35). Notably, forced suppression of PLZF results in higher frequencies of mouse iNKT1 cells, and the microRNA, Let7, is required for the downregulation of PLZF in mouse iNKT1 cells (47, 50). Thus, PLZF expression is essential for mouse iNKT cell development.

## Development of human iNKT cells

While the developmental pathway of mouse iNKT cells has been well studied (reviewed in (27, 51)), the exploration of human iNKT cell development has proven challenging. The earliest human iNKT cell progenitors emerge in the fetal thymus at 13 weeks of gestation and start to colonize peripheral tissues, especially the gut, but also the spleen and mesenteric lymph nodes in the second trimester (52, 53). In humans, the frequency of fetal thymic iNKT cells negatively correlates with gestational age and early studies failed to detect clear populations of iNKT cells in the postnatal thymus (52, 54). This observation implied that a significant portion of iNKT cell thymic development occurs during early fetal life and led to the initial hypothesis that the postnatal pool of iNKT cells is reconstituted by peripheral expansion of fetal-derived iNKT cells (52, 54). However, subsequent studies identified iNKT cells from human postnatal thymus, conclusively demonstrating that iNKT cells could be generated after fetal development (55, 56). The discrepancy between these studies may be partially attributed to the development of stringent detection reagents that facilitate the detection of these rare thymic progenitor cells (55, 56).

**Figure 2:**
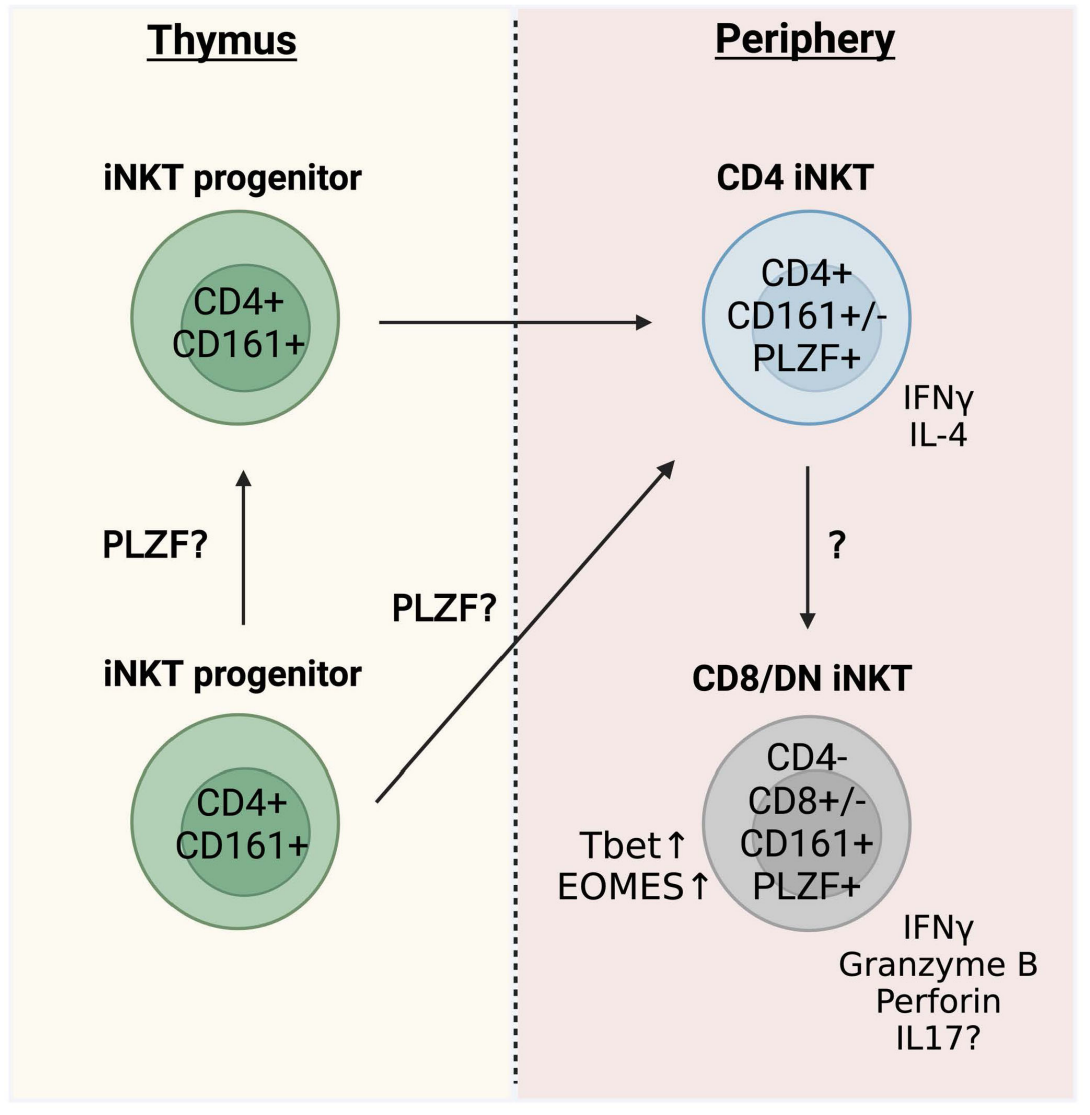
Human thymic iNKT cell development. Proposed pathway of human thymic iNKT cell development.

The timing of thymic iNKT cell development in humans and mice is different. While clear evidence of fetal thymic iNKT cells exists for humans, thymic development of iNKT cells in mice appears to be limited to the postnatal thymus (57). Specifically, iNKT cells fail to develop in neonatally thymectimized mice, revealing that the postnatal thymus is essential for mouse iNKT cell development (57). iNKT cells arise from the same lymphoid progenitors as conventional αβ-T cells but diverge at the CD4+CD8+ double-positive (DP) stage (58–60). In mice, selection of iNKT cells depends on CD1d expressed by DP thymocytes (61), and the presence of similar immature iNKT progenitors in both human and mouse thymus suggests that human iNKT cells are selected in a similar manner. Human studies investigating thymic maturation based on phenotypic heterogeneity demonstrate a dominance of CD4+ iNKT cells in the neonatal thymus, with CD4− iNKT cells accumulating in the blood with age (52, 55, 56, 62). Specifically, DN and CD8 iNKT cells do not emerge in peripheral blood until 6 months of age, although their levels in the thymus remain low even in older individuals (55). The scarcity of CD4− iNKT cells among thymic progenitors raises the question of the ontogenic origin of these cells (**Figure 2**) (55, 56). While the possibility exists that CD4− iNKT cells develop at a distinct extrathymic site, the lower T-cell receptor excision circle (TREC) content in these peripheral cells suggests that small numbers of CD4− iNKT cells exported from the thymus may increase in frequency following peripheral expansion, while CD4+ iNKT cells are maintained by thymic output (56). However, other studies could not find evidence of active proliferation in any peripheral iNKT cell subset outside of the thymus (55). A recent study analyzed iNKT TCR beta clonotypes in longitudinal matched samples and found hints of a linear relationship in which CD4+ cells give rise to CD4− subsets in the periphery but not *vice versa* (**Figure 2**) (11). The clear distinction in CD4 expression between thymic and peripheral iNKT cells is unique to humans, because even though the majority of mouse thymic iNKT cells are CD4+, there is a significant proportion of CD4− cells that emerge during development (63, 64).

Like mouse NKT1 cells, the human marker for mouse NK1.1 (CD161) is upregulated on fully mature human NKT cells. Although some CD161+ iNKT cells are already present in the thymus, the majority of human thymic iNKT cells appear to be CD161 negative, and CD161 expression accumulates with age in the periphery (52, 55, 56). Importantly, the increase in CD161+ iNKT cells was restricted to the blood (55). These data indicate that the developmental pathway of iNKT cells in humans is characterized by the upregulation of CD161 (21, 55). In accordance with this, a recent study used single-cell RNA sequencing to reveal the transcriptome of human thymic iNKT cells (65). Although the authors use of cryopreserved thymocytes might skew the ratio of developmental stages due to the preferential death of DP thymocytes, a profound upregulation of CD161 transcripts was identified in the clusters that comprised iNKT cells of later developmental stages (65, 66). Furthermore, a developmental trajectory was proposed that resembled murine iNKT cell development in which human immature “NKT2-like” cells develop into fully matured cells with a NKT1 signature (65).

Development of iNKT cell progenitors appears strongly dependent on IL-7 signaling since thymic and neonatal but also adult peripheral CD4+ iNKT cells express high levels of CD127 and are hyperresponsive to IL-7 (52, 62). CD4− iNKT cells from adult blood on the other hand express higher levels of CD122 and depend on IL-15 signaling (56).

Although the activated memory phenotype that is characteristic of human iNKT cells is imprinted during thymic development and already present in neonatal blood (67, 68), thymic iNKT cell progenitors do not seem to be functionally mature. This was indicated by the absence or comparatively weak cytokine response when stimulated *ex vivo*, especially when TCR ligation was used instead of PMA and ionomycin (23, 56, 62, 67). The observation that human iNKT cells are subject to substantial peripheral maturation was previously underpinned by a study recently published as a preprint that discovered shared transcriptional states between thymic naïve conventional T cells and iNKT cells using single-cell RNA-sequencing (69). Notably, the paper found no clusters of human iNKT cells that directly corresponded to mouse iNKT1, iNKT2, and iNKT17 subsets, suggesting that human iNKT cells may not fully differentiate into these distinct lineages, but rather acquire a mixed iNKT1/NKT17 effector program in the thymus (69). Similarly, data from Legoux, Lantz, and colleagues also support the idea of human iNKT cells acquiring a mixed Th1/17 effector program akin to the development of MAIT cells (70). Interestingly, only a small subset of thymic iNKT cells exhibited an effector signature, and this signature closely resembled that of iNKT cells in the peripheral blood of adult donors, whereas most thymic iNKT cells appeared to reside in a naïve state (69). It was suggested that the naïve phenotype of human thymic iNKT cells results from positive selection on thymic epithelial cells (TECs) instead of DP thymocytes, since TECs do not provide SLAM signaling, which is critical for the induction of the effector program in iNKT cells (69).

Overall, the cumulative data suggest that iNKT cells in the human thymus are CD4+ and CD161−. Thereby, the expression of CD161 is considered a major control point in the maturation of iNKT cells (21), which mostly occurs extrathymically and increases with age and reaches the highest levels in adult PBMCs. CD4− iNKT cell subsets (DN and CD8+) emerge later and almost exclusively in the periphery. The origin of these cells is still unclear but CD4− cells likely arise from CD4+ cells (**Figure 2**) (11).

## Transcriptional regulation of human iNKT development

PLZF is highly expressed in human peripheral iNKT cells (35, 36), although protein expression has not been examined for human iNKT cells from the thymus. A recent paper used single-cell RNA-sequencing to indicate that PLZF is expressed by mature subsets of human thymic iNKT cells, although gene expression appeared low (70). Interestingly, a patient with a biallelic mutation of PLZF revealed a failure to develop DN and CD161+ iNKT cells, consistent with perturbed thymic NKT cell development (71). These data correspond well with mouse studies (27, 35, 36, 46, 47) and strongly hint towards an equally important role of PLZF in the development of human iNKT cells (**Figure 2**) (71). It is not known if the dynamics of PLZF expression in humans is similar to the mouse and whether it is differentially expressed during the emergence of distinct functional subsets. Characterizing the expression of PLZF during human iNKT cell thymic development will help to definitively establish a role for PLZF in human iNKT cell development and its impact on lineage commitment.

We know from mouse studies that iNKT cell development is a tightly regulated process that relies crucially on signaling via the SLAM–SAP–Fyn axis, reviewed in (27). Consistent with this, the lack of SAP in patients with X-linked lymphoproliferative syndrome (XLP) correlates with an absence of iNKT cells (72, 73). Multiple other factors exist that are affected in those with primary immunodeficiencies and are associated with defects in iNKT and CD1-restricted T cells, reviewed in (74). Two factors that appear essential for human iNKT development are the Wiskott–Aldrich syndrome protein (WASp) and X-linked inhibitor-of-apoptosis (XIAP). WASp is mutated in Wiskott–Aldrich syndrome patients who exhibit an almost-complete absence of circulating iNKT cells (75). Mutations in XIAP can also cause XLP, and XIAP-deficient patients had low levels of iNKT cell levels, comparable to those seen in SAP-deficient XLP donors (76). The underlying mechanism from a developmental point of view is unexplored, but data from peripheral blood suggest that XIAP counteracts a proneness to increased apoptosis in iNKT cells mediated by PLZF, which may explain the abrogated iNKT cell development in XLP patients with a XIAP deficiency (77).

## Overlap between NKT cell development and other human unconventional T-cell pathways

We have recently characterized the thymic development of human MAIT cells and Vγ9Vδ2+ γδ T cells (27, 46, 78–80). Both pathways describe a linear three-stage thymic pathway that involves the upregulation of CD161. The earliest stage for human thymic MAIT cells is CD27−CD161− stage 1 cells that upregulate CD27 to become stage 2 CD27+CD161− cells before finally upregulating CD161 to become stage 3 CD27+CD161+ cells (46, 78, 80). For human thymic Vγ9Vδ2+ γδ T cells, stage 1 cells are CD4+CD161− that transition to become CD4−CD161− stage 2 cells and then to CD4−CD161+ stage 3 cells (79). Importantly, the thymic development of both MAIT cells and Vγ9Vδ2+ γδ T cells involves the upregulation of PLZF as cells mature from stage 1 to stage 3. Furthermore, both pathways involve major changes in the expression of transcription factors, surface molecules, chemokines, chemokine receptors, and cytotoxic killing granules as cells mature from stage 1 to stage 3. For example, *CD1A*, *CD4*, *CCR9, LEF1, TCF7, BACH2, BCL11B*, and *SOX4* are expressed by stage 1 MAIT and Vγ9Vδ2+ γδ T cells and are downregulated as these cells mature to their stage 3 counterparts (78, 79). These studies highlight clear overlap between the thymic development pathway for human MAIT cells and Vγ9Vδ2+ γδ T cells (78, 79); thus, it will be important to establish if these characteristics of development among these unconventional T-cell subsets extends to the development of human iNKT cells.

Despite the strong overlap in the development of human MAIT cells and Vγ9Vδ2+ γδ T cells, particularly at early stages, some important differences were observed in the degree of maturation of stage 3 subsets from each population. For example, stage 3 thymic MAIT cells produced very little cytokines compared to mature MAIT cells from the blood, suggesting that significant extrathymic development is required for human MAIT cells, and this was supported by RNA-sequencing data (46, 78, 80). In contrast, stage 3 Vγ9Vδ2+ γδ T cells from human thymus produced levels of IFNγ and TNFα upon stimulation similar to their matched blood counterparts (79). Furthermore, transcriptomic analysis revealed very few differences in the genes expressed by CD4−CD161+ stage 3 cells from the thymus and matched blood, suggesting only minor extrathymic development for human Vγ9Vδ2+ γδ T cells (79). As discussed above, very few thymic human iNKT cells express CD161 and acquisition of CD161 occurs predominantly in periphery (52, 55, 56). These data indicate that human MAIT cells and iNKT cells require additional development steps in the periphery that are yet to be fully defined.

Transcriptomic analysis is a powerful approach to dissect the complexities of human unconventional T-cell development (65, 69, 70, 78, 79), although validation of proposed pathways using phenotypic analysis, functional assays, and precursor-product experiments is crucially required for investigating the development of iNKT cells.

## Challenges to deciphering a human thymic iNKT developmental pathway

Even though iNKT cells can be detected in the human thymus and there are similarities between mouse and human thymic iNKT development, our knowledge of the developmental pathway in humans is incomplete. A major challenge in defining the pathway for human iNKT cell development is their relative scarcity within thymus tissue. Their frequency in the human thymus is far lower than in mice and approximately 100 times lower than in matched human peripheral blood, which hinders detailed analysis (23). As demonstrated by Berzins et al., detection of an extremely rare cell population in the human thymus requires refined flow cytometry protocols and stringent gating to reliably detect them above background levels (55). The technological advance in high-dimensional full spectrum flow cytometry has significantly expanded and now allows for a more comprehensive phenotyping of rare cell populations (81–84). Furthermore, the use of MACS enrichment of MAIT cells and Vγ9Vδ2+ γδ T cells was critical to our efforts to define the development pathways for these cells from human thymus (46, 78, 79). The integration of MACS technology in combination with advances in flow cytometry and transcriptomics approaches like RNA-seq will likely provide significant new insights into the development of human thymic iNKT cells (85).

## How human immunotherapies could benefit from understanding a defined developmental pathway for NKT cells

Since their discovery more than 30 years ago, an ever-growing body of evidence has emerged that human iNKT cells represent a promising therapeutic target for autoimmune disorders, GvHD, and cancer immunotherapy, and as vaccine adjuvants for infectious diseases (86–90). This is based on their potential for off-the-shelf use in immunotherapies (17), and their rapid, multifunctional effector responses. Importantly, iNKT cells express an invariant TCR shared by all individuals that confers common lipid antigen specificity, and moreover, CD1d is monomorphic, which abolishes any concerns caused by highly polymorphic MHC molecules. Hence, a detailed knowledge about iNKT subsets, their function, and especially how these subsets develop will be important to successfully utilize them for distinct therapeutic approaches (91). For example, understanding what genes are switched on and off during the development of cytokine and cytotoxic granule producing iNKT cells could be exploited to develop NKT cell-based therapies with a desired functional output.

Out of several therapeutic options for iNKT cells, iNKT-based tumor immunotherapies are an especially promising field for future therapeutic applications. Reduced numbers of iNKT cells were observed not only in autoimmunity or obesity (18, 92, 93), but also in several human malignancies [reviewed in 94, 95]. Infiltration of IFNγ-producing iNKT cells thereby positively correlated with better survival (96), demonstrating the importance of iNKT cells in the tumor setting. Furthermore, iNKT cells have been shown to mediate potent anti-tumor immunity (14, 97, 98) by a range of different mechanisms like direct cytotoxicity (99, 100), lysis of tumor promoting immune cell subsets (101), or creating a tumor suppressive immune environment by stimulation of NK cells or CD8 T cells via IFNγ release and DC maturation, respectively (96, 102).

Indeed, many clinical trials have been conducted that employed various strategies to stimulate NKT cells to boost their numbers or induce their function with the objective of targeting different cancers (103, 104). While iNKT cell immunotherapy was feasible and well tolerated in most of these trials, there was only partial success in eradicating the tumors. The reasons for this are not clear, but the pan-targeting strategies employed in most of the trials may be a factor. Anti-tumor activity is dependent on a strong type 1 immunity and the release of IFNγ (105). Notably, iNKT cells are not exclusively pro-inflammatory—and thus anti-tumorigenic—but also mediate potent anti-inflammatory immune responses (9) mediated by IL-4 that could promote tumor growth (10, 106). These conflicting iNKT functions are believed to be regulated through the context in which the cells are activated and on the presence of heterogeneous subsets. These factors include the mode of activation (i.e., cytokine- versus ligand-driven activation), the microenvironment (i.e., the presence of cytokines or antigen-presenting cells), and the strength of TCR signaling (8, 107).

As mentioned earlier, iNKT cell subsets elicit heterogeneous immune responses in which anti-tumor functions appear to be primarily mediated by the CD4-negative subset (10, 13, 14). This suggests that trials might need to target specific subsets of iNKT cells, although recent evidence has shown that relying solely on the expression of CD4 to define the anti-tumor potential of iNKT subsets is not enough. Subset differences were demonstrated in a work by Tian et al. that showed superior anti-tumor potential of CD62L+ CAR-iNKT cells, compared with CD62L− CAR-iNKT cells (108). More recently, a circulating NKT cell subpopulation was identified in humans that exhibited pronounced cytotoxic properties that was marked by the expression of CXCR6 and CD244 (109). Investigation of the developmental trajectory of the murine equivalent to these cells showed that they were occupying a separate developmental niche in the thymus. Development of these CXCR6+CD244+ cells was highly dependent on IL-15 in the thymus, whereas “conventional” iNKT cells depend more on IL-15 in the periphery (109). Efforts to generate human iNKT cells for off-the-shelf cancer immunotherapy using human CD34+ stem cells transduced with NKT TCR and cultured in artificial thymic organoids successfully resulted in hematopoietic stem cell (HSC)-engineered iNKT cells of high purity and yield (110). Most HSC-iNKT cells exhibited a CD4+CD8+ DP phenotype after 6 weeks of culture, before further developing into CD8+ or CD4−CD8− DN iNKT cells, but not CD4+ iNKT cells, after 10 weeks of culture (110). Collectively, these studies have important implications for the *in vitro* manipulation and expansion of specific subsets of iNKT cells for immunotherapy, highlighting how detailed knowledge of iNKT subset development helps to identify subpopulations suitable for therapeutic approaches.

## Concluding remarks

A crucial advance in our efforts to utilize NKT cells in immunotherapies will be to identify functionally distinct subsets and characterize their development pathways. This will help enable subsets with the desired functions to be selectively targeted, ensuring different subsets do not oppose each other’s functions (111). Important unknowns include whether there is direct thymic imprinting of human NKT cell subsets as there is in mice, and determining whether the functions of human iNKT cells are polarized in the thymus or periphery, and understanding what drives this process (i.e., specific stimuli) (112). The frequency of iNKT cells is another important consideration given their low frequency in human blood and tissues. The use of transcriptomics to study the development of unconventional T cells has greatly increased our understanding of the molecular requirements to produce functional subsets of T cells. Therefore, uncovering the origin of mature human iNKT cell subsets and defining a more detailed thymic developmental pathway may then allow the specific targeting of iNKT cell subsets in new-generation immunotherapies that exploit the unique characteristics of iNKT cells.
